# New insight into inter-organ crosstalk contributing to the pathogenesis of non-alcoholic fatty liver disease (NAFLD)

**DOI:** 10.1007/s13238-017-0436-0

**Published:** 2017-06-22

**Authors:** Xu Zhang, Xuetao Ji, Qian Wang, John Zhong Li

**Affiliations:** 1 Department of Biochemistry and Molecular Biology, School of Basic Medical Sciences, Nanjing Medical University, Nanjing, 211166 China; 2 Jiangsu Province Key Laboratory of Human Functional Genomics, Nanjing Medical University, Nanjing, 211166 China

**Keywords:** non-alcoholic fatty liver disease, hepatic lipid metabolism, hypothalamus, gut-liver axis, adipose tissue

## Abstract

Non-alcoholic fatty liver disease (NAFLD) is the most common cause of chronic liver dysfunction and a significant global health problem with substantial rise in prevalence over the last decades. It is becoming increasingly clear that NALFD is not only predominantly a hepatic manifestation of metabolic syndrome, but also involves extra-hepatic organs and regulatory pathways. Therapeutic options are limited for the treatment of NAFLD. Accordingly, a better understanding of the pathogenesis of NAFLD is critical for gaining new insight into the regulatory network of NAFLD and for identifying new targets for the prevention and treatment of NAFLD. In this review, we emphasize on the current understanding of the inter-organ crosstalk between the liver and peripheral organs that contributing to the pathogenesis of NAFLD.

## Introduction

NAFLD refers to excess fat accumulation in the liver of patients with no history of alcohol abuse or other causes of secondary hepatic steatosis. Clinically, it represents a complex spectrum of hepatic damage, from simple steatosis and nonalcoholic steatohepatitis (NASH), progressing to fibrosis, and ultimately cirrhosis (Anstee et al., 2011). NAFLD is one of the most common public health problems worldwide. Recent epidemiology studies suggest that NAFLD is present in 12%–38% of the general population and NASH affects 3%–15% (Vernon et al., 2011). In China, NAFLD is becoming a greater health concern, with increasing rates of metabolic disturbances, such as obesity, type 2 diabetes mellitus, and dyslipidemia. The prevalence of NAFLD in patients with type 2 diabetes is 28%–55% and in those with hyperlipidemia is 27%–92% (Fan and Farrell, 2009). NAFLD is regarded as a hepatic manifestation of metabolic syndrome and is strongly associated with obesity and insulin resistance (Stojsavljevic et al., 2014). Although NAFLD is strongly associated with obesity and insulin resistance, its pathogenesis has not been fully elucidated and therapeutic options are limited. Current treatment is focused on the control of the disease process and risk factors. Thus a comprehensive understanding of the pathogenic mechanism of the development of NAFLD is extremely important (Cohen et al., 2011).

The underlying mechanism for the development and progression of NAFLD is complex and multifactorial. Different theories have been proposed; initially, the “two hits” hypothesis was developed to explain the pathogenesis of the NAFLD spectrum (Day and James, 1998). According to this traditional doctrine, the factors that metabolically promote the deposition of triacylglycerides (TAG) in the liver, including a high-fat diet, obesity, and insulin resistance, represent the “first hit” in the pathogenesis of NAFLD. Signaling process such as extracellular cytokines, adipokines, bacterial endotoxin, mitochondrial dysfunction, and/or endoplasmic reticulum (ER) stress provide the second hit for progression to NASH, activating inflammatory cascades and fibrogenesis (Greenberg et al., 1991; Peverill et al., 2014). However, the traditional “two-hit” pathophysiological theory has been challenged as knowledge of the interplay among insulin resistance, adipokines, adipose tissue inflammation, and other less recognized pathogenic factors have recently increased. In particular, it has been suggested that hepatic steatosis represents an epiphenomenon of several distinct injurious mechanisms, rather than a true “first hit” (Bulankina et al., 2009). For this reason, the initial “two-hit” theory explaining the progression from NAFLD to NASH has been evolved into the “multiple parallel hits” hypothesis (Jou et al., 2008; Buzzetti et al., 2016).

**Figure 1:**
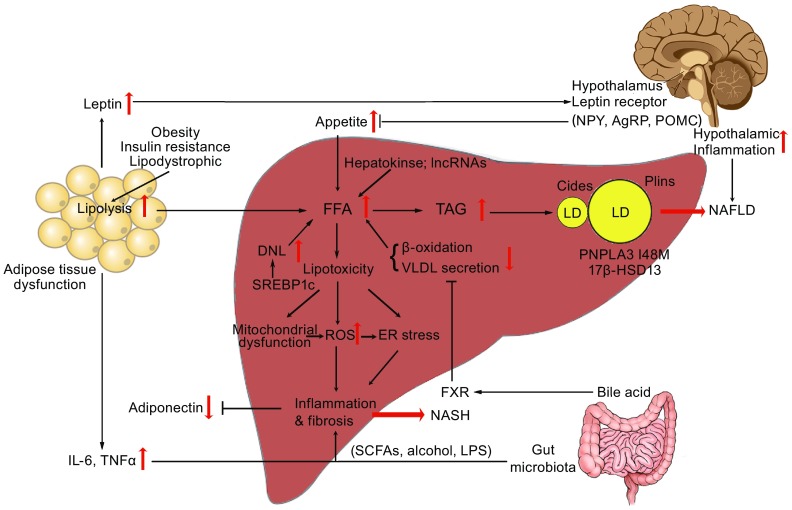
**The “cross-talk” between liver and peripheral organs in the pathogenesis of NAFLD**. The impairment of the hypothalamic signaling pathway due to mutations (*Leptin receptor and MC4R*) by affecting the appetite or inflammation leads to the development of obesity and NAFLD. Dysfunction of adipose tissue in obesity, lipodystrophy or insulin resistance provides a source of excess fat and release of adipokines such as *Leptin*, *Adiponectin*, *Resistin*, and proinflammatory cytokines such as TNF-α and IL-6 that participated in the pathogenesis of NAFLD. In addition, emerging evidence suggests that an altered gut permeability consequently affect circulating levels of molecular such as LPS, FFA, bile acid, and to the release of pro-inflammatory cytokines by the regulation of TLR and FXR further influence the development and progression of NAFLD, recognized as effect of gut–liver axis. In the liver, the dysregulation of lipid *de novo* lipogenesis and imbalance of lipid influx and efflux causes lipotoxicity and may result in mitochondrial dysfunction, overproduction of ROS and ER stress as well as the consequent activation of inflammatory responses, thus influencing the risk of progression of NAFLD to NASH, as observed in obesity and insulin resistance

Several lines of evidence suggest that continuous inter-organ crosstalk sustains all processes involved in NAFLD pathogenesis, and crucial roles of the gut, hypothalamus, adipose tissue, and intestine have been suggested. In this review, we emphasize on the current understanding of the inter-organ crosstalk between the liver and peripheral organs that participated in the pathogenesis of NAFLD (Fig. 1).

## Liver and NAFLD

As mentioned above, epidemiological studies have revealed that NAFLD is a specific manifestation of metabolic syndrome and is strongly associated with obesity and insulin resistance. Specifically, hepatic steatosis arises from an imbalance between TAG influx and efflux. During hepatic TAG formation, fatty acids are derived from the diet, *de novo* lipogenesis (DNL), and adipose tissue via lipolysis. Once in hepatocytes, free fatty acids (FFAs) undergo acyl-CoA synthetic activity to form fatty acyl-CoAs, which further be oxidized in mitochondria via the β-oxidation pathway, re-esterified to TAG, and stored in lipid droplets (LDs) or coupled to apolipoproteins and further secreted as very low-density lipoprotein (VLDL) (Cohen et al., 2011). In obese patients, the increased efflux of FFAs from adipose tissue to the liver may induce defects in the insulin signaling pathway and contribute to insulin resistance (Bandsma et al., 2008; Braliou et al., 2008). In states of insulin resistance, sterol regulatory element binding protein-1c (SREBP-1c), the master regulator of DNL, is over-expressed and DNL is up-regulated (Stefan et al., 2008). Studies in rodent models revealed a peculiar feature of hepatic insulin resistance, in which hepatic glucose metabolism becomes unresponsive to insulin but hepatic lipogenesis continues unabated, recognized as selective hepatic insulin resistance (Brown and Goldstein, 2008, Li et al., 2010b, Cook et al., 2015). Selective hepatic insulin resistance has been proposed to explain the common clinical phenotype of hyperglycemia, hyperlipidemia, and NAFLD in T2D patients (Vatner et al., 2015). Additionally, β-oxidation of FFAs is inhibited in insulin resistance states, thus further promoting hepatic lipid accumulation (Donnelly et al., 2005, Postic and Girard, 2008, Cohen et al., 2011, Lambert et al., 2014). The inhibition of TAG incorporation into new VLDL by blocking micorsomal TAG transport protein (MTTP) and apolipoprotein B (ApoB) causes impaired TAG secretion which induces TAG accumulation in the liver (Amaro et al., 2010, Di Filippo et al., 2014). Hepatic inactivation of diacylgycerol acyltransferase 2 (DGAT2), a key enzyme catalyzing TAG synthesis, also reduces the hepatic TAG content and subsequently increases FFA oxidation, resulting in the worsening of steatohepatitis in mouse models (Yamaguchi et al., 2007).

Lipid droplets (LDs) are dynamic cytoplasmic organelles found ubiquitously in cells. They are linked to many cellular functions, including lipid storage for energy generation and membrane synthesis, viral replication, and protein degradation (Walther and Farese, 2012). Interestingly, connections between LD-associated proteins and NAFLD have been recently identified through genome-wide association studies (GWAS) as well as genomic and proteomic studies (Zhang et al., 2017).

Perilipins (PLINs) were the first type of specific LDs marker proteins identified in 1991 (Greenberg et al., 1991). The PLIN family contains several members, including perilipin 1 (PLIN1), perilipin 2/adipophilin (PLIN2), perilipin 3/Tip47 (PLIN3), perilipin 4 (PLIN4), and perilipin 5/OXPAT (PLIN5). While are not essential for LDs formation, PLINs are important for the regulation of lipid metabolism (Bulankina et al., 2009). PLIN1 is undetectable in normal liver, but is expressed in the liver of humans with NAFLD (Straub et al., 2008). PLIN2 has negative regulatory effects on VLDL lipidation (Chang et al., 2010) and TAG secretion. PLIN2, PLIN3, and PLIN5 levels are elevated in fatty liver of humans and their ablation alleviates steatosis in mouse models (Imai et al., 2007, Wang et al., 2015). Cell death-inducing DFFA-like effector (CIDE) proteins, which are located on LDs and the ER, are involved in fatty liver progression. Cidea and Cidec are responsible for liver steatosis under fasting and obese conditions by mediating the fusion of small and large LDs (Gong et al., 2011, Xu et al., 2016), while Cideb promotes lipid storage under a normal diet by regulating the process of VLDL lipidation and LDs fusion in the liver (Li et al., 2007, Ye et al., 2009)

Patatin-like phospholipid domain-containing protein 3 (PNPLA3) is another ER- and LD-associated protein. It is one of the few examples of a protein that has been validated in several populations to be conclusively shown to be associated with NAFLD, particularly the I148M (rs738409 C/G) variant (Romeo et al., 2008, Anstee, 2015). As a member of the PNPLA family, PNPLA3 is most closely related to PNPLA2 (ATGL), the major cellular TAG lipase. Neither ablation nor overexpression of wild-type PNPLA3 affects the liver fat content in mice, whereas transgenic mice with hepatic specific overexpression of human 148M or PNPLA3 I148M knock-in mice exhibit increased hepatic TAG contents and LD sizes and develop hepatic steatosis (Basantani et al., 2011, Li et al., 2012, Pirazzi et al., 2012, Smagris et al., 2015). Moreover, PNPLA3 I148M affects VLDL secretion in rat hepatoma cells and mouse livers (Pirazzi et al., 2012). These observations suggest two possible mechanisms for the pathogenesis of NAFLD induced by PNPLA3 mutations. First, PNPLA3 may alter lipolysis, not by hydrolysis activity itself, but by the inhibition of other proteins in the family, like ATGL (Smagris et al., 2015). Second, PNPLA3 mutations may reduce the mobilization of TAG on LDs (Pirazzi et al., 2012). Further studies are required to determine the precise mechanisms by which PNPLA3 regulates of hepatic lipid metabolism and determines its association with NASH and fibrosis.

17β-Hydroxysteroid dehydrogenase 13 (17β-HSD13) is a hepatic LD protein associated with NAFLD identified in recent proteomic studies (Su et al., 2014). Another independent study confirmed this result and also indicated a slight upregulation of 17β-HSD13 in patients with NASH without fatty liver (Kampf et al., 2014). In a study of fasted and refed mice, 17β-HSD13 expression was markedly higher on hepatic LDs of mice in the high-fat diet group than on those of mice in the low-fat group (Crunk et al., 2013). Overexpression of 17β-HSD13 in a mouse hepatocyte cell line induced liver steatosis and lipid accumulation. It also lead to increased expression of proteins involved in lipid synthesis, such as mature SREBP-1 and FAS, suggesting that 17β-HSD13 is involved in NAFLD development by promoting lipogenesis (Su et al., 2014).

In recent years, in the pathogenesis of NAFLD, more and more attention has been paid to the hepatokines, which are mainly produced by the liver. NAFLD seems to be associated with altered hepatokines production such as fetuin-A, fibroblast growth factor-21 (FGF-21), selenoprotein P, sex hormone-binding globulin (SHBG), angiopoietin-related growth factor (AGF) and leukocyte derived chemotaxin 2 (LECT2) (Lebensztejn et al., 2016). There is a suggestion that fetuin-A constitutes a link between obesity, insulin resistance and NAFLD, plays a major pathogenic role in metabolic disease (Mori et al., 2011, Pal et al., 2012, Stefan and Haring, 2013). Meanwhile, FGF21 has recently emerged as a novel hormone, leading to beneficial effects on glucose metabolism and lipid homeostasis, in addition to promoting rapid body weight loss in rodents (Li et al., 2010a). A number of publications showed significantly higher serum concentrations of FGF-21 in a population of patients with NAFLD compared to the controls (Yilmaz et al., 2010a, Yilmaz et al., 2010b, Giannini et al., 2013).

In addition, microRNAs (miRNAs), short, noncoding RNAs that regulate gene expression, have been associated with histological features of NAFLD and are readily detected in the circulation. As such, miRNAs are emerging as potentially useful noninvasive markers with which to follow the progression of NAFLD (DiStefano and Gerhard, 2016). Not only the potential mechanistic role of miRNAs involved in the pathogenesis of NAFLD described elsewhere (Gerhard and DiStefano, 2015), but also the multicellular nature and pathophysiological progression of NAFLD suggested that miRNAs may be associated with different disease stages (DiStefano and Gerhard, 2016). In a study of 84 circulating miRNAs measured in 47 NASH patients, 30 individuals with simple steatosis and 19 healthy controls, levels of miR-122, miR-192, and miR-375 were upregulated in patients with NASH compared to those with simple steatosis, and were associated with histological disease severity (Pirola et al., 2015). Furthermore, another non-coding RNA, long non-coding RNAs (lncRNAs) have emerged as important regulatory molecules in the pathogenesis of NAFLD (Li et al., 2015, Chen, 2016). Several fatty liver-related lncRNAs (FLRLs) have been identified to be related to lipogenesis, such as FLRL8, FLRL3 and FLRL7, through proteins in PPAR signaling pathway, such as Fabp5, Lpl and Fads2, indicating their potential regulatory role in lipid metabolism (Chen et al., 2017).

Fat accumulates in the liver of patients with NAFLD mainly in the form of TAG. This accumulation occurs concurrently with an increase in lipotoxicity owing to high levels of FFAs, free cholesterol, and other lipid metabolites. This lipotoxicity is believed to further lead to mitochondrial dysfunction with oxidative stress and the production of reactive oxygen species (ROS) and ER stress-associated mechanisms (Malhotra and Kaufman, 2007, Vonghia et al., 2013, Schneider and Cuervo, 2014, Buzzetti et al., 2016). Mechanistically, alterations in the structure and function of mitochondria contribute to the pathogenesis of NAFLD. Mitochondrial dysfunction may collapse respiratory oxidation with the impairment of fat homeostasis, generation of lipid-derived toxic metabolites, and overproduction of ROS (Begriche et al., 2006). Consequentially, increased ROS causes not only oxidative stress damage, but also the activation of Kupffer cells, whose activation is a key step in the development of NASH (Vonghia et al., 2013). Furthermore, ROS accumulation and related changes in autophagy cause chronic ER stress, which is closely related to the apoptosis of hepatocytes (Malhotra and Kaufman, 2007).

## Hypothalamus and NAFLD

The central nervous system is crucial for the regulation of energy metabolism. In particular, the hypothalamus has critical roles in sensing and integrating signals from the periphery tissue and effecting appropriate physiological changes to maintain metabolic homeostasis (Zoccoli et al., 2011). The arcuate nucleus (ARC) provides many physiological roles involved in feeding, metabolism, and cardiovascular regulation (Bouret et al., 2004, Coppari et al., 2005, Sapru, 2013). More specifically, the ARC of hypothalamus is a specific nuclear group to sense different peripheral indicators of metabolic status and integrates responses to afferent information to control food intake and body weight (Schwartz et al., 2000).

The best-characterized peripheral indicator is leptin, which is an adipokine produced primarily in visceral adipocytes. Leptin signaling in the hypothalamus regulates hunger and energy expenditure, which are mediated by a neural circuitry comprising orexigenic and anorectic signals (Kwon et al., 2016). ARC neurons produced anorexigenic neuropeptides pro-opiomelanocortin (POMC), the precursor of α-melanocyte-stimulating hormone (α-MSH), and cocaine-amphetamine-regulated transcript (CART) are both activated by leptin (Cone et al., 1996, Kristensen et al., 1998), whereas orexigenic neuropeptide Y (NPY) and Agouti-related peptide (AgRP) are inhibited by leptin and activated by ghrelin (Broberger et al., 1998, Hahn et al., 1998). Central administration of NPY increases food intake, inhibits the thyroid axis, and decreases sympathetic nervous system outflow to brown adipose tissue, thus lowering energy expenditure. Conversely, stimulation of α-MSH receptors suppresses food intake, activates the thyroid axis, and increases energy expenditure (Wynne et al., 2005).

The spontaneous leptin mutation model *ob*/*ob* mice develop severe diabetes with marked hyperglycemia and a propensity to overeat, resulting in obesity and the development of hepatic steatosis (Mayer et al., 1951). A study reported that an obese leptin-deficient girl with hepatic steatosis exhibited rapid improvement after the introduction of recombinant leptin therapy (von Schnurbein et al., 2013). A recent meta-analysis indicated that circulating leptin levels were higher in patients with NAFLD than in controls. Importantly, such increased leptin levels were consistent with the severity of NAFLD, and the association remains significant after the exclusion of pediatric or adolescent populations as well as morbidly obese individuals subjected to bariatric surgery (Polyzos et al., 2016). Leptin action in peripheral tissues involves interaction with specific transmembrane receptors (leptin receptor, LEPR). The observation that LEPR is associated with NAFLD has been pointed by several studies. Among patients with NAFLD, LEPR polymorphisms were found to be associated with lipid metabolism, obesity parameters, and insulin resistance (Aller et al., 2012, Zain et al., 2013). Recent study suggested that the combined effect of variants of LEPR and PNPLA3 conferred increased susceptibility to NAFLD (Zain et al., 2013).

Approximately 20% of ARC NPY neurons innervate the paraventricular nucleus (PVN) (Baker and Herkenham, 1995). Stimulation of this pathway leads to increased food intake through direct stimulation of NPY receptors Y1R and Y5R and through AgRP antagonism of melanocortin receptors MC3R and MC4R in the PVN (Woods et al., 1998; Simpson et al., 2009). Moreover, the administration of α-MSH into the PVN inhibits food intake and the orexigenic effect of NPY administration (Woods et al., 1998). MC4R-KO mice fed with a high-fat diet developed a liver condition similar to human NASH and further progressed to HCC, which is associated with obesity, insulin resistance, and dyslipidemia. These phenotypes seem to result from a loss of function of MC4R in the hypothalamus, rather than in the liver itself (Itoh et al., 2011). Polymorphisms in *MC4R* are associated with alanine aminotransferase and BMI (Guan et al., 2014), but not with hepatic fat content in population-wide studies (Haupt et al., 2009).

In addition, hypothalamic inflammation was also shown involvement in the regulation of hepatic steatosis in physiological and pathophysiological conditions (Milanski et al., 2012; Valdearcos et al., 2015). Obesity-associated hypothalamic inflammation was first reported in a rat model of diet-induced obesity (De Souza et al., 2005), which was further confirmed by other groups (Zhang et al., 2008, Kleinridders et al., 2009, Milanski et al., 2009, Ozcan et al., 2009, Posey et al., 2009, Holland et al., 2011a). The observation that genetic interventions that disrupt neuronal inflammation can block both obesity and hypothalamic leptin resistance during feeding with a high-fat diet supports such role of inflammation in NAFLD pathogenesis. Several signaling pathways of the innate immune system have been identified as candidate mediators of hypothalamic inflammation during high-fat diet feeding, including toll-like receptor 4 (TLR 4), c-Jun N-terminal kinase (JNK), suppressor of cytokine signaling 3 (SOCS3), and pro-inflammatory cytokines, as well as the induction of ER stress and autophagy defects (De Souza et al., 2005, Zhang et al., 2008, Kleinridders et al., 2009, Milanski et al., 2009, Ozcan et al., 2009, Posey et al., 2009, Holland et al., 2011a).

## Adipose and NAFLD

Adipose tissue, in addition to its function as the major storage depot for TAG, is an active endocrine organ that senses metabolic signals and secretes hormones that profoundly influence hepatic lipid metabolism (Berg et al., 2001). Excess adipose tissue in obesity and a lack of adipose tissue in the lipodystrophic state are associated with insulin resistance and NAFLD (Kahn and Flier, 2000).

Obesity, especially visceral adiposity, is a major risk factor for NAFLD in humans. Adipose tissue is a source of FFAs that are delivered to the liver, used for TAG synthesis by hepatocytes, and released into the blood (Diehl et al., 2005). The excess fat storage in obese and insulin-resistant individuals, and increased lipolysis in adipose tissue, are important source of FFA for hepatic TAG formation and storage in the liver of NAFLD patients (Cusi, 2012, Bril et al., 2014).

Additionally, NAFLD is also a typical finding in lipodystrophic patients. Lipodystrophy syndromes represent extreme and opposite ends of the adiposity spectrum related to obesity. A selective loss of body fat is the hallmark of lipodystrophy syndromes associated with an increased prevalence of insulin resistance in the skeletal muscles and the liver, increased plasma TAG levels, and hepatic steatosis (Kahn and Flier, 2000; Agarwal et al., 2004). In patients with lipodystrophy, defective adipose tissue is unable to store even regular amounts of energy. The inability of adipose tissue to store lipid in the form of TAG results in ectopic fat accumulation in aberrant tissues, such as the liver and skeletal muscle. Consequentially, excess TAG deposition in the liver (hepatic steatosis) and skeletal muscle induces NAFLD and peripheral insulin resistance (Agarwal and Garg, 2006). Several genetic mutations are strongly associated with lipodystrophy, including mutations in *Agpat2*, *Pparγ*, *Lmna*, *Zmpste24*, *Akt2*, and *Bscl2* (Agarwal and Garg, 2006). Among these mutants, AGPAT2 induced lipodystrophy was indicated either by reducing triglyceride accumulation in adipocytes or levels of glycerophospholipids and hence affecting adipocyte function (Agarwal et al., 2004).

As an endocrine organ, adipose tissue is responsive to both peripheral and central metabolic signals by secreting a number of proteins, termed adipokines, to execute a variety of local, peripheral, and central effects (Stojsavljevic et al., 2014). For example, leptin is such a peptide hormone secreted mainly by visceral adipocytes to modulate food intake, body fat composition, insulin sensitivity, thermogenesis, and the immune system via the hypothalamus, as described above (Stojsavljevic et al., 2014).

Another adipokine, adiponectin, is a soluble matrix protein, is produced by visceral adipocytes (Arita et al., 1999). A great number of studies using human, animal, and *in vitro* models to investigate the pathogenesis and molecular mechanisms have shown that adiponectin influences obesity, insulin resistance, NAFLD, and other components of metabolic syndrome (Hu et al., 1996, Arita et al., 1999, Ryo et al., 2004, Matsuzawa, 2010). Adiponectin circulates in the serum in several oligomeric isoforms whose specific effects have been observed (Schober et al., 2007, Wang et al., 2008). In addition to that adiponectin improves hepatic and peripheral insulin resistance, it also presents some anti-inflammatory and hepato-protective activities (Kadowaki et al., 2006). These effects are partly achieved by enhancing the deacylation of ceramide sphingolipids, independently of AMPK, especially in hepatocytes (Holland et al., 2011b). The anti-inflammatory effects are achieved by blocking the activation of NF-κB, secreting anti-inflammatory cytokines, and inhibiting the release of pro-inflammatory cytokines, such as TNF-α and IL-6 (Tilg and Moschen, 2006). Adiponectin was also shown to have a direct antifibrotic effect (Kamada et al., 2003), which could be mediated by the activation of AMPK (Adachi and Brenner, 2008). Enhanced liver fibrosis has been demonstrated in mice lacking adiponectin (Kamada et al., 2003) whereas the delivery of recombinant adiponectin significantly improves steatohepatitis in mice (Xu et al., 2003). The antioxidant effects of adiponectin are mediated by its receptor AdipoR1 and thus decreased adiponectin levels in obesity may have causal roles in mitochondrial dysfunction and insulin resistance (Kamada et al., 2003). In obese patients, reduced adiponectin and increased leptin levels may result in hepatic steatosis and the activation of inflammation and fibrogenesis (Tsochatzis et al., 2006). Interestingly, adiponectin has been proposed as a good predictor of the necroinflammatory grade and fibrosis in NAFLD (Musso et al., 2005, Handy et al., 2010, Polyzos et al., 2011, Finelli and Tarantino, 2013). Furthermore, serum levels of adiponectin are reduced in obese subjects with type 2 diabetes mellitus and insulin resistance (Statnick et al., 2000, Maeda et al., 2001, Weyer et al., 2001, Spranger et al., 2003, Ozcelik et al., 2013). Replenishment of adiponectin ameliorates insulin resistance and glucose intolerance and decreases the liver triglyceride content in mice (Berg et al., 2001, Fruebis et al., 2001, Yamauchi et al., 2001, Okada-Iwabu et al., 2013). Additionally, resistin, another adipocyte-derived polypeptide, was initially found to be upregulated in obesity and insulin resistance (Holcomb et al., 2000, Steppan et al., 2001). In NAFLD, its levels were also higher than those in controls and were positively correlated with liver inflammation and fibrosis severity, although this result remains controversial (Zou et al., 2005, Pagano et al., 2006, Tsochatzis et al., 2008). *In vitro* studies have suggested that resistin participates in the progression of inflammation and its pro-inflammatory effects may be mediated by activating the c-Jun-N-terminal kinase (JNK) and NF-κB pathways (Zhang et al., 2010).

Currently, some view that development of NAFLD and insulin resistance may be resulted from imbalance cytokines, namely, increased pro-inflammatory and decreased anti-inflammatory cytokines (Diehl et al., 2005, Day, 2006). For instance, TNF-α is a proinflammatory cytokine that has various biological effects, including metabolic inflammatory and proliferative effects. It exhibits increased expression levels in the liver and adipose tissue of subjects with NAFLD. Data from human and animal studies have indicated that TNF-α has a role in the development of NAFLD and is a predictor of NASH that is correlated with advanced stages (Diehl, 2004, Cai et al., 2005, Jarrar et al., 2008). Furthermore, large quantities of IL-6 are secreted by visceral fat than by subcutaneous fat in obese individuals (Fontana et al., 2007). The role of visceral fat as an independent factor associated with NAFLD is due in large part to its secretion of proinflammatory cytokines (Van der Poorten et al., 2008). Inflammation and fibrosis in NAFLD patients were associated with increased systemic IL-6 (Van der Poorten et al., 2008), which decreased by therapy with Vitamin E in NAFLD patients in a small pilot study (Kugelmas et al., 2003).

## Gut and NAFLD

Recently, compelling evidence links the gut microbiome, intestinal barrier integrity, bile acid and NAFLD, indicating that interactions between the liver and the gut, the so-called “gut–liver axis” may play a critical role in NAFLD onset and progression.

Emerging evidence indicates that the human gut microbiota is involved in the development of obesity and related complications, including NAFLD (Drenick et al., 1982, Backhed et al., 2004, Zhu et al., 2015). Gut microflora may stimulate hepatic fat deposition and promote NASH by several mechanisms (Aron-Wisnewsky et al., 2013, Gkolfakis et al., 2015). Changes to the microbiome regulate gut permeability and increase hepatic exposure to injurious substances that increase hepatic inflammation and fibrosis. The first evidence of an increased intestinal permeability (leaky gut) and tight junction alterations in NAFLD patients compared with healthy subjects was reported in 2009 (Miele et al., 2009). Since then, more studies in humans and mice have confirmed the association between impaired intestinal barrier function and hepatic fibrogenesis and inflammation (Miele et al., 2009, Gabele et al., 2011).

The gut microbiota also regulates immune balance and participates in the development and homeostasis of overall host immunity (Burcelin et al., 2012). The cross-talk between host and bacteria, which depends on Toll-like receptors (TLRs) or NOD-like receptors, is responsible for innate and adaptive immune responses that protect the host and maintain intestinal homeostasis (Compare et al., 2012). TLRs recognize highly conserved microbial molecules called “pathogen-associated molecular patterns” (PAMPs) or damage-associated molecular patterns (DAMPs) and initiate a signaling cascade leading to the activation of pro-inflammatory genes, such as *TNF-α*, *IL-6*, *IL-8*, and *IL-12* (Pisetsky, 2011, Alisi et al., 2012). Lipopolysaccharide (LPS), the most extensively studied PAMP, is a component of the gram-negative bacteria cell membrane and the active component of endotoxin. LPS-TLR-4 signaling activation is related to insulin-resistance and NASH. Studies on TLR-4 null mice have confirmed that TLR-4 is essential for hepatic fat deposition and NASH development (Poggi et al., 2007, Saberi et al., 2009, Henao-Mejia et al., 2012). In addition, the inflammasome, that is composed of leucine-rich-repeat-containing proteins and nucleotide-binding domain (NLRPs) can act as sensors of PAMPs and DAMPs and participate in the activation of lipid peroxidation and ROS production during NAFLD/NASH progression (Thuy et al., 2008, Harte et al., 2010, Henao-Mejia et al., 2012). Moreover, dysbiosis also affects the metabolism of food substrates, by increasing the production of certain short-chain fatty acids and alcohol and depleting choline.

The bile acid 90% excreted by the gallbladder is reabsorbed in the small intestine and is recycled back to the liver through the portal vein and have emerged as relevant signaling molecules that function in the liver to regulate lipid and carbohydrate metabolic pathways as well as energy homeostasis. Bile acids may function as signaling molecules via a variety of receptors, including members of the nuclear receptor superfamily (farnesoid X receptor [FXR; NR1H4], Vitamin D receptor [NR1I1], and pregnane X receptor [NR1I2]) and members of the G-protein-coupled receptor superfamily (TGR5), to regulate their own synthesis as well as other metabolic processes, such as glucose, lipid, and energy homeostasis (Maeda et al., 2001). Specifically, FXR, originally named for its ability to bind to farnesoid, has shown to play a role in the regulation of lipid metabolism (Sinal et al., 2000, Schaap et al., 2014, Carr and Reid, 2015, Mazuy et al., 2015, Fuchs et al., 2016). FXR-KO mice exhibit a proatherogenic lipoprotein profile with markedly elevated serum and hepatic cholesterol and triglycerides levels (Sinal et al., 2000, Arab et al., 2017). The activation of FXR represses hepatic DNL and stimulates fatty acid β-oxidation, limiting hepatic lipid accumulation (Pineda Torra et al., 2003, Watanabe et al., 2004, Savkur et al., 2005, Moore, 2012). FXR can also promote plasma VLDL triglyceride clearance by inducing the expression of *ApoCII*, an activator of lipoprotein lipase, and suppressing the expression of *ApoCIII*, an inhibitor of lipoprotein lipase activity (Mazuy et al., 2015, Fuchs et al., 2016). In addition, the gut flora modifies bile acid metabolism and FXR/TGR5 signaling and hence contributes indirectly to the development of NAFLD (Tremaroli et al., 2012).

The colon is a major site of gut bacterial fermentation, yielding high levels of short chain fatty acids (SCFAs, 70–130 mmol/L) (Duncan et al., 2009). The main substrates for the production of SCFAs by the colonic microbiota are dietary carbohydrates that have escaped digestion in the small intestine, collectively referred to as dietary fibre (Psichas et al., 2015). Experiments comparing the feces of obese and lean individuals demonstrated that the level of short-chain fatty acids was higher in the obese whereas residual calories from food were concomitantly reduced (Turnbaugh et al., 2006, Schwiertz et al., 2010). SCFAs therefore have been proposed to contribute to obesity and liver steatosis as they provide approximately 10% of daily caloric consumption and may enhance nutrient absorption by promoting expression of glucagon-like peptide 2 (den Besten et al., 2013, Boursier and Diehl, 2015) (Zhu et al., 2015). However, SCFAs also improve lipid and glucose metabolism and maintain intestinal homeostasis (den Besten et al., 2013, Puertollano et al., 2014, Boursier and Diehl, 2015). Hence, the net effect of SCFAs on NAFLD pathogenesis remains unclear and is likely complex. For example, although total cecal SCFA concentrations of recipient mice given flora from responder versus nonresponder mice were similar in the Leroy study, two branched-chain fatty acids (isobutyrate and isovalerate) were significantly higher in responder-receiver mice (Boursier and Diehl, 2015). Branched-chain fatty acids, which can be *de novo* synthesized by several gut bacterial species, have been associated with insulin resistance and metabolic disease development (Newgard, 2012, Boursier and Diehl, 2015).

## Extracellular vesicles and NAFLD

Exosomes are small membrane-bound extracellular vesicles (EVs) released by various types of cells into biological fluids (Sato et al., 2016). There are two main populations of EVs, namely exosomes and microparticles (MPs), which differ in size, composition, and mechanism of generation. Exosomes are small, 30–100 nm in diameter, and are released by exocytosis as a result of multivesicular bodies fusing with the plasma membrane (Masyuk et al., 2013). EVs have been the topic of great interest in recent years in NAFLD research. Patients with NAFLD or NASH secrete increased levels of microvesicles derived from macrophages and natural killer T cells (Kornek et al., 2012). Another EV study has shown that the expression levels of various proteins within vesicles are enhanced in a mouse model of NAFLD, and that protein expression pattern differs between exosomes and microvesicles (Povero et al., 2014).

EVs are involved in NAFLD pathology because they regulate cell-cell communication and a number of pathophysiological events in various types of cells via horizontal transfer of their cargo including proteins (membrane, cytosolic, and nuclear), RNAs (including mRNAs and microRNAs), and lipids (Yuan et al., 2009, Diehl et al., 2012, Raposo and Stoorvogel, 2013). Notably, released EVs do not only stay in the tissue of origin, but also circulate in the blood stream (Povero et al., 2014). Recent studies have demonstrated that primary and immortalized hepatocytes are capable of producing and releasing both exosomes and MPs (Conde-Vancells et al., 2008, Witek et al., 2009, Pan et al., 2012, Povero et al., 2013). EVs are formed and released during the accumulation of lipotoxic lipids in hepatocytes, which is a key mechanism of liver damage and disease progression in NAFLD (Povero et al., 2014).

In obese individuals, adipocyte-derived exosomes are known to contribute to the development of insulin resistance via activation of adipose-resident macrophages and secretion of proinflammatory cytokines that can result in insulin resistance (Deng et al., 2009). Recent evidence indicated that visceral obese adipocytes shed exosomes that contain mediators capable of activating end-organ inflammatory and fibrotic signaling pathways and these exosomes contain miRNAs capable of regulating end-organ TGF-β and Wnt/β-catenin signaling in obesity-related comorbid conditions (Zhu et al., 2015).

## Conclusion

The pathogenesis of NAFLD and its progression is a complex process. Increasing evidence indicates that a number of diverse and parallel processes contribute to the development of NAFLD and liver inflammation. The impairment of the hypothalamic signaling pathway due to mutations or inflammation leads to the development of obesity and NAFLD. Dysfunction of adipose tissue in obesity or lipodystrophy provides a source of excess fat and results in the secretion of multiple factors involved in the pathogenesis of NAFLD. In addition, emerging evidences suggest that an altered gut microbiota can influence the development and progression of NAFLD, possibly via the gut–liver axis. In the liver, the dysregulation of lipid *de novo* lipogenesis and imbalance of lipid influx and efflux causes lipotoxicity which may further result in mitochondrial dysfunction and ER stress as well as the consequent activation of inflammatory responses, as observed in obesity and insulin resistance. Combined with the rapidly increasing expending field of studies of miRNAs and LncRNA research suggest that the identification and validation these non coding RNA may improve the diagnosis and clinical monitoring of NAFLD progression.

An improved knowledge of the pathogenic “cross-talk” between the liver and extra-hepatic organs will not only help to modulate known risk factors associated with the onset of NAFLD and/or its progression to end-stage liver disease but may also provide insight for the development of new pharmacological treatments for NAFLD.
